# Impact of Alcohol Content on Alcohol–Ester Interactions in Qingxiangxing Baijiu Through Threshold Analysis

**DOI:** 10.3390/foods14244290

**Published:** 2025-12-12

**Authors:** Huan Zhang, Liuyan Zheng, Kaixuan Zhu, Tianxu Liu, Lexuan Yang, Lijuan Ma, Xin Zhang, Lin Yuan, Liping Du

**Affiliations:** Key Laboratory of Industrial Fermentation Microbiology Ministry of Education, College of Biotechnology , Tianjin University of Science and Technology, Tianjin 300457, China; zhanghuan90@tust.edu.cn (H.Z.); 16629068097@163.com (L.Z.); m15102663248@163.com (K.Z.); 15124012153@163.com (T.L.); 19948130094@163.com (L.Y.); malj@tust.edu.cn (L.M.); dsss666@126.com (X.Z.)

**Keywords:** high- and reduced-alcohol, alcohol and ester, interaction relationship, olfactory threshold, Qingxiangxing baijiu

## Abstract

Alcohols and esters are core flavor-active constituents of Qingxiangxing Baijiu (QXB), yet ethanol concentration’s regulatory role in their thresholds and interactions remains unclear. Physicochemical analysis showed reduced-alcohol QXB (L-QX, 42%, *v*/*v*) had higher total acid (1.48 g/L) but lower total ester (1.52 g/L) than high-alcohol QXB (H-QX, 53%, *v*/*v*; 1.20 g/L total acid, 2.05 g/L total ester). Sensory evaluation (0–5 scale) revealed H-QX had higher fruity (3.6 vs. 2.0), grassy (3.2 vs. 1.8), and grainy (3.0 vs. 1.9) aroma scores, while L-QX showed higher sour (2.1 vs. 1.5) and lees (1.7 vs. 1.1) notes (*p* < 0.05). The quantification of gas chromatography-flame ionization detection (GC-FID) determined the concentrations of eight alcohols and esters in H-QX samples and identified that most flavor compounds had higher concentrations than L-QX samples. Three alternative forced-choice tests showed 53% ethanol elevated olfactory thresholds (OTs) of five compounds, with ethyl lactate (1.53-fold) and isopentanol (1.89-fold) vs. 42%. For 16 alcohol–ester binary mixtures, 12 pairs had OT ratios (53% vs. 42%) < 1, especially 3 pairs (e.g., n-propanol-ethyl acetate) < 0.5. OAV/S curve analyses indicated all 16 mixtures had masking effects, with 11 pairs stronger at 42%. Verification validated 53% ethanol mitigated masking, enhancing fruity/grassy aromas by 38.1%/25.0%. This study provides support for QXB dealcoholization flavor regulation.

## 1. Introduction

Chinese Baijiu, a traditional distilled liquor in China, has developed twelve flavor types over thousands of years due to variations in brewing environments, techniques, and other factors [1]. Among these, Qingxiangxing (QXB), nongxiangxing (NXB), and jiangxiangxing Baijiu are the three most popular among consumers. JXB is typified by “exquisite aroma, rich taste and long-lasting aftertaste”, while NXB features “intense pit aroma, smooth sweetness, harmonious flavor, clean taste and long-lasting after-taste”. Compared with other types, QXB meets contemporary consumer drinking demands with its “pure clean aroma, mellow-sweet softness and refreshing finish [2,3,4].” This Baijiu (Chinese liquor) is produced using grain as the raw material, with Daqu, Xiaoqu, Fuqu, and Jiuqu (yeast) as saccharifying and fermenting agents. The manufacturing process consists of solid-state fermentation in vessels (e.g., jars, pits), followed by solid-state distillation, aging, and blending. Critically, neither edible alcohol nor any color, aroma, or flavor substances that are not endogenously generated via its own fermentation are added to the product, either directly or indirectly [5].

As an important category of Chinese Baijiu, QXB enjoys a high reputation both domestically and internationally [6]. The style of Baijiu is shaped by these flavor substances and their interactions. More than 730 volatile substances have been identified in QXB, including esters and alcohols [7]. The content and proportion of the four major esters in Baijiu are among the important factors affecting the typical style and aroma quality of the liquor body. For instance, a significant decrease in the ratio of ethyl hexanoate to ethyl lactate will result in a shorter and weaker aroma of the liquor body, inhibition of the main aroma, and thus an uncoordinated aroma [8,9,10]. Ethyl acetate and ethyl lactate are key contributors to the floral and fruity aromas in QXB. Ethyl butyrate is present in low concentrations in QXB, yet it remains a key aroma-active component. Alcohols in QXB are not only important aroma-presenting substances but also play a crucial role in the taste and aroma coordination of Baijiu. There are a wide variety of alcohols in Baijiu, such as n-propanol, n-butanol, isobutanol, n-pentanol, and isopentanol. These higher alcohols possess unique aromas and flavors; although their content in Baijiu is low, they play an important modifying role in the flavor of Baijiu [11,12,13]. Specifically, isopentanol and isobutanol impart a mellow body and certain grassy notes [14]. The interaction between aroma substances is essential for the expression of Baijiu style. Zhou, H. et al. [15] demonstrated that through the Feller additive model and odor activity value (OAV) method, the binary mixtures formed by 1-propanol and four kinds of esters in a 53% ethanol-water system exhibit a masking effect. However, the interactions between other higher alcohols and esters in QXB remain unclear so far.

Since alcohol poses certain hazards to the human body, and as consumption patterns evolve and market demands diversify, low-alcohol Baijiu with lower alcohol volume fraction has attracted increasing attention from consumers [16,17,18]. However, low-alcohol Baijiu suffers from defects such as insufficient aroma. Current studies have shown that this phenomenon is partially attributed to the low solubility of flavor substances in low-alcohol solutions [19], which reduces the perception of flavor. On the other hand, low alcohol content can lower the threshold of flavor substances, which is beneficial to flavor perception [18,20]. Therefore, the underlying reason for the reduced flavor in low-alcohol Baijiu still needs further investigation. Baijiu is a complex system, and the interactions between aroma substances can significantly alter the threshold of binary or multivariate aroma substances, which exerts an undeniable impact on the flavor expression of Baijiu. Then, the insufficient flavor of reduced alcohol Baijiu is caused by the altered overall threshold in the complex system, leading to weakened perception. At present, research on the effect of different alcohol contents on the threshold of flavor substances only focuses on the threshold of single compounds, while studies on the effect of alcohol content on the overall threshold of binary compounds have not been reported yet.

Baijiu quality evaluation depends on the synergy of physicochemical analysis and sensory evaluation, with existing interdisciplinary studies advancing flavor research primarily through three core approaches [21,22]. First, physicochemical and chromatographic analysis quantifies key components: Li et al. [23] linked total acid/ester change rates to product grades across 28–50% ethanol; Zhang et al. [24] identified 32 volatile compounds with higher concentrations in high-alcohol NXB via gas chromatography flame ionization detection (GC-FID); Luo et al. [25] combined GC with total acid/ester attenuation to assess low-alcohol QXB storage quality. Second, sensory evaluation reveals flavor differences: Zhang et al. [26] compared high/low-alcohol JXB via sensory analysis, identifying variations in acidic, floral, and fruity notes; Peng et al. [27] integrated sensory evaluation and chromatographic analysis to confirm lower skeleton components (e.g., ethyl caproate) in low-alcohol Wuliangye (35–39% vs. 45–52%). Third, interaction analysis (e.g., S-curve, OAV) explores flavor dynamics, such as additive/synergistic effects between ethyl isobutyrate and ethyl isovalerate [28]. However, most studies focus on NXB and JXB, leaving high/reduced-alcohol QXB (21–69% ethanol [29]) understudied, highlighting the value of this work.

Based on this, the present study investigates two QXB samples with different alcohol contents (42% and 53%) through modern analytical techniques combined with sensory evaluation methods. The research systematically proceeds as follows: first, physicochemical analysis, sensory evaluation, and electronic nose analysis are employed to examine differences in aroma profiles between the two samples; subsequently, chromatographic techniques such as GC-FID are used to quantitatively analyze their major aroma compounds; particular emphasis is placed on studying the influence of ethanol concentration on the thresholds of eight key alcohol and ester compounds and the thresholds of sixteen binary mixtures; finally, the S-curve method and OAV analysis are applied to explore interaction mechanisms within alcohol–ester binary systems under varying ethanol concentrations. This approach aims to elucidate the chemical basis of how alcohol strength affects flavor perception in QXB, thereby providing a scientific foundation for product development and quality enhancement of Baijiu with different alcohol contents.

## 2. Materials and Methods

### 2.1. Sample Collection

Two samples of QXB of the same brand but different alcohol contents were purchased from the market (produced by Shanxi Xinghuacun Fenjiu Co., Ltd., Fenyang, China), designated as L-QX (where “L” indicates an alcohol content of 42%) and H-QX (where “H” indicates an alcohol content of 53%), respectively. Both products use water, sorghum, barley, and peas as raw materials. The production process of H-QX involves diluting the distilled high-alcohol Baijiu to reduce its alcohol content, which then becomes L-QX. Ethanol aqueous solution simulated system samples with alcohol contents of 42% and 53% were prepared, consisting of 16 groups of single-compound systems and 32 groups of alcohol–ester pairwise binary mixtures. The single compounds and binary mixtures were composed of the following reagents: isobutanol, isopentanol, n-propanol, n-butanol, ethyl hexanoate, ethyl acetate, ethyl butyrate, and ethyl lactate. The alcohol contents of both the Baijiu samples and the ethanol aqueous solutions of the simulated systems were 42% and 53%.

### 2.2. Reagents

The following external standards were used in GC-FID analysis, isobutanol, isopentanol, n-propanol, n-butanol, ethyl formate, ethyl hexanoate, ethyl octanoate, ethyl benzoate, diethyl malonate, diethyl succinate, and acetaldehyde (purity > 99.5%) were purchased from Merrell Chemical Technology Co., Ltd. (Shanghai, China), and ethyl acetate, ethyl valerate, ethyl leucinate, methanol, active amyl alcohol, n-hexanol, 2-phenylethanol, propionic acid, isobutyric acid, isovaleric acid, and furfural (purity > 99.5%) were purchased from Aladdin Technology Company (Shanghai, China). Ethyl butyrate, acetic acid, isoamyl acetate, ethyl 2-methylbutanoate, and lactic acid (purity > 99.5%), as well as ethyl lactate (purity > 99%) were purchased from Macklin Company (Shanghai, China). Ethyl tridecanoate and acetoin (purity > 99.5%) were purchased from Shanghai Yuanye Bio-Technology Co., Ltd. (Shanghai, China); ethyl decanoate, ethyl isovalerate, ethyl nonanoate, 2,3-butanediol, acetal, and isobutyraldehyde (purity > 99.5%) were purchased from Sigma-Aldrich (Shanghai, China). Ethanol aqueous solution was refined by Tianjin Guangfu Fine Chemical Research Institute (Tianjin, China).

### 2.3. Physicochemical Analysis

Total acids were determined in accordance with GB 12456-2021 “National Food Safety Standard—Determination of Total Acids in Foods” [30], and total esters and alcohol content were measured following GB 10345-2022 “Method of Analysis for Baijiu [31].”

Determination of alcohol content: Pipette 50 mL of commercially available Baijiu into a clean graduated cylinder, allow it to stand for several minutes to stabilize air bubbles. Subsequently, immerse an alcohol hydrometer into the sample, wait for 5 min until stabilization to take the reading, and record the corresponding temperature for conversion.

### 2.4. Sensory Evaluation

#### 2.4.1. Descriptive Analysis of the Aroma Profiles

Following the method described in the previous literature [32], a sensory evaluation panel was established, comprising 10 experienced evaluators with proficient olfactory discrimination abilities. All participants signed informed consent to participate. Experiment has passed ethical review, and proof was provided in Table S1. This panel conducted quantitative descriptive analysis (QDA) of the aroma profiles of QXB. Sensory evaluations were performed under controlled environmental conditions, with a constant room temperature of 25 °C. Through preliminary screening and consensus discussion, seven key aroma attributes were identified: floral, fruity, sweet, grassy, grainy, lees, and sour [29]. The sensory attributes and their corresponding reference standards are presented in Table 1. A 0–5 point intensity scale was adopted for scoring the seven aroma attributes (0: no perceptible aroma, 5: extremely intense aroma). To ensure reproducibility, all evaluations were performed in triplicate.

#### 2.4.2. Electronic Nose Analysis

An electronic nose (E-nose) system equipped with ten distinct sensors was employed for analysis. The operational conditions for the E-nose analysis were adapted from the method described by Zhou et al. [33]. For sample preparation and analysis, 10 μL of the Baijiu sample was pipetted into a 200 mL wide-mouth vial, which was immediately sealed with plastic wrap to prevent volatile loss. The sealed vial was then incubated at 25 °C for 3 min to equilibrate the headspace. Subsequently, the E-nose sampling needle was inserted into the vial for headspace sampling. The detection environment was maintained at a constant temperature of 25 °C, with the following key parameters set: carrier gas flow rate = 45 mL/min, sampling flow rate = 45 mL/min, analysis duration = 100 s, self-cleaning duration = 180 s, sample preparation time = 5 s, and sampling interval = 1 s (to stabilize the baseline signal). Each sample was analyzed in triplicate to ensure the reproducibility of the results.

### 2.5. GC-FID Analysis

Volatile compound analysis in Baijiu followed Fan et al. [34] with minor modifications, using GC-FID (Agilent Technologies, Santa Clara, CA, USA). Samples were direct injection. Due to varying GC-FID sensitivities across compound classes, distinct chromatographic columns were employed for accurate analysis. Identification in QXB utilized retention time matching; quantification employed external standard calibration. Calibration method was used for quantitative determination of compound concentrations.

GC analysis employed an HP-INNOWAX column (30 m × 320 μm × 0.25 μm, Agilent Technologies, Santa Clara, CA, USA) under the following conditions: injector at 250 °C; carrier gas N_2_ (99.999%) at 0.8 mL/min constant flow; split ratio 10:1. The oven program was 35 °C (hold 1 min), ramp to 50 °C at 3 °C/min, to 90 °C at 5 °C/min, to 125 °C at 25 °C/min (hold 3 min), to 230 °C at 5 °C/min (hold 2 min); total runtime 41.4 min. Calibration curves were generated by plotting analyte concentration against peak area using standard solutions.

Analysis used a CP-WAX57-CB capillary column (50 m × 250 μm × 0.2 μm, Agilent Technologies, Santa Clara, CA, USA) with the following parameters: 1 μL injection volume; 30:1 split ratio; injector at 250 °C; carrier gas (99.999% N_2_) flow at 1 mL/min; FID at 260 °C. The oven temperature program was 35 °C (hold 1 min), ramp to 70 °C at 3 °C/min, then to 190 °C at 3.5 °C/min (hold 22 min; total runtime 37.6 min). Samples were analyzed via direct liquid injection. Quantification employed external standard calibration curves (analyte concentration vs. peak area).

### 2.6. Determination of Lactic Acid Content by HPLC

HPLC analysis followed Emanuela et al. [35] with modifications. Lactic acid in QXB was quantified using an Agilent 1260-VWD system (UV detector; SilGreen H column: 70 m × 0.78 mm × 8 μm; Agilent Technologies, Santa Clara, CA, USA). Sample preparation: Two samples were diluted 10-fold, filtered (0.22 μm organic membrane), transferred to 2 mL vials, and stored pending analysis. Chromatographic conditions mobile phase B: 97.5% H_3_PO_4_ (aqueous solution; pH 2.15)/2.5% MeOH; mobile phase C: MeOH; flow rate: 0.5 mL/min; injection: 10 μL; column: 80 °C; detection: 210 nm; gradient: 0 min (100% B); 13 min (100% B); 15 min (5% B : 95% C); 18 min (5% B); 20 min (100% B); 30 min (100% B). Lactate was identified by standard retention times and quantified via external standard calibration.

### 2.7. Determination of OT

Eight target compounds—isopentanol, isobutanol, n-butanol, n-propanol, ethyl acetate, ethyl lactate, ethyl hexanoate, and ethyl butyrate—were selected. These were mixed pairwise at H-QX concentrations, yielding 16 binary mixtures. Each mixture was prepared in 42% and 53% ethanol solutions for threshold determination. Olfactory detection thresholds for individual compounds and binary mixtures were determined via the 3-AFC method (ISO 13301:2018 [36]). Assessors and conditions followed Table S1. Each analyte was tested at 10 concentrations. Detection probability (*p* = correct identifications/total panelists) was calculated for individual compounds and mixtures. *p*-values were corrected using Equation (1). All experiments were performed in triplicate.

(1) P=(3∗p-1)/2

Olfactory detection thresholds (individual compounds and pairwise mixtures) were calculated using the S-curve method, a statistically based approach recognized for precise sensory threshold determination. A characteristic S-shaped relationship exists between logarithmic concentration (log C) and detection probability (P). Using Origin 2024 software, fitting was performed on the dataset derived from 3-AFC experiments in accordance with the S-shaped curve Equation (2)and drew an S-shaped fitting curve of concentration log C detection probability P (correlation coefficient R > 0.95). In Equation (2): P = corrected detection probability; x = log C; X_0_ = logarithmic threshold; D = S-curve slope. The measured threshold concentration corresponds to P = 0.5. Detection probability P(AB) was determined via the 3-AFC method.

(2) $$P=\frac{1}{1+e^{-\frac{x-x0}{D}}}$$

The theoretical detection probability of mixtures, P(AB), was calculated using Feller’s summation equation [37], as shown in Equation (3). P(A) and P(B) represent the measured corrected detection probabilities of components A and B at their respective concentrations. A theoretical log (concentration)-P curve was generated and fitted to the S-curve Equation (2) to obtain the theoretical S-curve. The theoretical threshold was defined as the concentration at P = 0.5.

(3) P(AB)=P(A)+P(B)-P(A)P(B)

### 2.8. OAV Analysis of Interactions Between Alcohols and Esters

The measured Odor Activity Value (OAV) of a mixture equals its total concentration divided by its experimentally determined olfactory threshold (3-AFC method). The theoretical OAV is the sum of individual component OAVs [38]. Aroma synergy between ester-alcohol pairs was determined using Equation (4): masking effect: X > 1; additive effect: 0.5 < X < 1; synergistic effect: X < 0.5 where X = theoretical OAV/measured OAV.

(4) $$X=\frac{OAV(A)+OAV(B)}{OAV(A+B)}$$

### 2.9. S-Curve Analysis of Interactions Between Alcohols and Esters

Binary mixture thresholds were determined experimentally (Section 2.7) and theoretically (Feller’s summation equation). Interaction types were classified by ratio D (Equation (5)), D = experimental threshold/theoretical threshold. The classification criteria for D are as follows. Synergistic effect: D ≤ 0.5; additive effect:0.5 < D < 1; No interaction: D = 1; masking effect: D > 1 [39].

(5) $$D=\frac{Experimental\;threshold}{Theoretical\;threshold}$$

### 2.10. Verification Experiment

Eight compounds—ethyl lactate, ethyl acetate, ethyl hexanoate, ethyl butyrate, isopentanol, isobutanol, n-butanol, and n-propanol—were added to L-QX based on H-QX concentration differences so that the concentrations of the eight compounds in the recomposed model (RM) were aligned with that of the H-QX sample. Sensory evaluation and electronic nose analyses followed Section 2.4 methods.

### 2.11. Statistical Analysis

Data processing and analysis were conducted using Microsoft Excel 2016, and all results are presented as the mean ± standard deviation (SD) based on three independent replicate analyses. Statistical analysis of the data was performed using SPSS Statistics software (Version 27.0, IBM Corp., Armonk, NY, USA), where one-way analysis of variance (ANOVA) was applied to assess differences among groups. A probability value of *p* < 0.05 was considered statistically significant. Graphical visualization, including radar charts, bar charts, and S-curves, was generated using OriginPro 2024 software (OriginLab Corp., Northampton, MA, USA).

## 3. Results and Discussion

### 3.1. Determination of Physicochemical Analysis of QXB

Physicochemical indices serve as the foundation for evaluating Baijiu quality, while sensory experience can intuitively reflect its flavor characteristics and quality grade. Table 2 demonstrated that the measured alcohol contents of L-QX and H-QX were 41.9% and 52.9%, respectively. Both samples met the quality criteria for premium-grade Baijiu, as their total acid contents were ≥ 0.5 g/L and total ester contents were ≥ 1.10 g/L. Specifically, the total acid content of L-QX was 1.48 g/L, which was higher than that of H-QX (1.2 g/L), while the total ester content of L-QX (1.52 g/L) was lower than that of H-QX (2.05 g/L). Liu et al. [40] conducted a quality analysis of NXB and reported that the mass concentration of total acids decreased with increasing ethanol volume fraction, whereas the mass concentration of total esters increased with rising ethanol volume fraction. This finding is consistent with the results of the present study: H-QX (with higher alcohol content) exhibited a lower total acid content but a higher total ester content compared to L-QX (with lower alcohol content).

### 3.2. Sensory Analysis of QXB with Different Alcohol Contents

**Figure 1:**
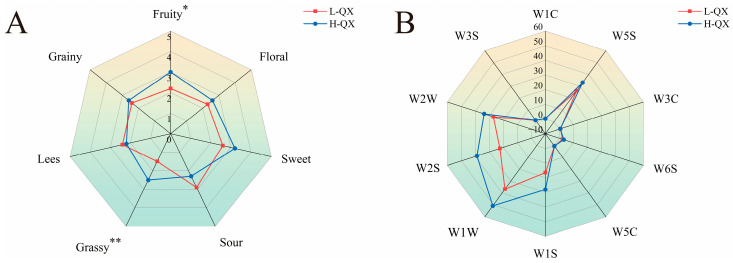
Aroma profiles of QXB with different alcohol concentrations based on radar chart of sensory evaluation (**A**) and E-nose (**B**). *, significant difference (*p* < 0.05); **, highly significant difference (*p* < 0.01).

To accurately elucidate the differences in the characteristics of QXB between the two types of alcoholic beverages, this study initially compared their aroma profiles through sensory evaluation experiments. Sensory evaluation revealed significant aroma profile differences between H-QX and L-QX Baijiu samples (Figure 1A), particularly in aroma type, intensity, and balance. H-QX showed higher scores for fruit, floral, sweet, grassy, and grain aromas, while exhibiting lower sour and lees notes than L-QX. Statistically significant differences in fruit and grassy aromas indicated alcohol content-dependent variations in sensory attributes. Furthermore, the E-nose has been demonstrated to effectively detect and analyze volatile compounds in samples, providing supplementary data to support these sensory findings [41]. Electronic nose analysis (Figure 1B) further supported these findings. Sensors W1S, W2S, W1W, and W2W showed higher response values for H-QX, confirming distinct volatile compound profiles. These findings demonstrate that the aroma profiles of both samples exhibit marked differences due to variations in alcoholic strength.

### 3.3. Analysis of Aroma-Active Compounds in QXB with Different Alcohol Contents

The distinct flavor compounds in Baijiu contribute to its diverse aroma profiles, with these components serving as critical determinants of its aroma characteristics, style, and overall quality [42]. Section 3.1 and Section 3.2 evaluated the differences in aroma characteristics between the two alcohol content variants through physicochemical analysis and sensory analysis, followed by qualitative and quantitative analysis of flavor compounds using chromatographic techniques—enabling comprehensive flavor profiling through integrated assessment of aromatic and compositional attributes. As shown in Table 3, GC-FID identified 36 aroma compounds (17 esters, 5 acids, 4 aldehydes, 1 ketone, 9 alcohols) across the two samples: the L-QX sample contained 26 compounds, the H-QX sample contained all 36, and 26 compounds were common to both, 9 esters, including ethyl acetate, ethyl butyrate, ethyl hexanoate, ethyl lactate, ethyl benzoate, ethyl leucineate, diethyl malonate, diethyl succinate, and ethyl tridecanoate, 9 alcohols, including methanol, n-propanol, isobutanol, n-butanol, active amyl alcohol, isopentanol, n-hexanol, 2,3-butanediol, 2-phenylethanol, 2 acids, acetic acid, isobutyric acid, ketones, 1 acetoin, and 4 aldehydes, including acetaldehyde, acetal, isobutyraldehyde, and furfural, were detected in two QXB. The quantitative analysis showed that most of the flavor substances in high-alcohol Baijiu were higher than those in reduced alcohol Baijiu. It can be inferred that the sensory differences observed in QXB samples with varying alcohol contents may be attributed to discrepancies in the types and concentrations of flavor compounds. Quantitative analysis revealed that ester and alcohol contents in high-alcohol baijiu were relatively higher compared to the reduced alcohol counterparts. Previous studies have demonstrated that esters and alcohols are, respectively, associated with fruity and grassy aromas [14], which may explain the prominence of these sensory attributes in high-alcohol Baijiu.

### 3.4. Determination of OT of Main Alcohol and Ester in Different Alcohol Contents

Thus, the critical question which emerges is whether different alcohol contents affect the sensory profile of Baijiu by modulating the perception of esters and alcohols. Therefore, we selected four primary esters (ethyl acetate, ethyl lactate, ethyl hexanoate, ethyl butyrate) and four key alcohols (isopentanol, n-propanol, n-butanol, isobutanol) as research targets and conducted their perceptual characteristics based on the concentrations of these substances in high-alcohol Baijiu. Investigating the effect of ethanol concentration on the threshold of aroma compounds is essential for a comprehensive understanding of changes in aroma perception [43]. First, thresholds of eight target compounds (n-butanol, isobutanol, isopentanol, n-propanol, ethyl acetate, ethyl lactate, ethyl hexanoate, ethyl butyrate) were determined. Subsequent analyses examined alcohol–ester binary mixtures to assess synergistic perceptual effects.

#### 3.4.1. The Effect of Ethanol Content on the OT of the Single Alcohol or Esters Compounds

**Figure 2:**
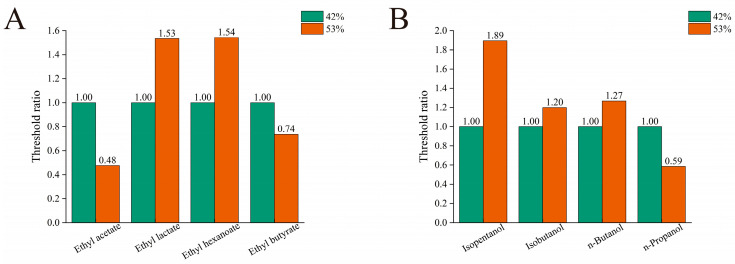
Threshold ratio for esters (**A**) and alcohols (**B**) at varying ethanol concentrations (with a threshold of 42% as 1).

Samples matching the substance content of the H-QX sample were prepared separately in 42% and 53% ethanol aqueous solutions for evaluation. The detection threshold measured in 42% ethanol aqueous solutions was set as the baseline (1.0). Thresholds obtained in 53% ethanol were then expressed as ratios relative to this baseline. Figure 2A shows that detection thresholds for ethyl lactate and ethyl hexanoate in 53% ethanol aqueous solutions increased significantly compared to those in 42% ethanol aqueous solutions, by approximately 1.53 folds and 1.54 folds, respectively. In contrast, thresholds for ethyl acetate and ethyl butyrate decreased in 53% ethanol, registering only 0.48 folds and 0.74 folds of their respective thresholds in 42% ethanol. This indicates notably lower detection thresholds for ethyl acetate and ethyl butyrate in the higher ethanol concentration. Figure 2B shows that thresholds for isopentanol, isobutanol, and n-butanol in 53% ethanol aqueous solutions increased relative to 42% ethanol aqueous solutions by 1.89 folds, 1.20 folds, and 1.27 folds, respectively. Conversely, only n-propanol exhibited a lower threshold in the higher ethanol concentration.

In summary, thresholds for ethyl lactate, ethyl hexanoate, isopentanol, isobutanol, and n-butanol were higher in 53% than in 42% ethanol aqueous solutions, whereas thresholds for ethyl acetate, ethyl butyrate, and n-propanol were lower. This pattern aligns with literature reports indicating lower thresholds for isobutanol, n-butanol, ethyl hexanoate, and ethyl lactate in 40% ethanol aqueous solutions compared to 50% ethanol aqueous solutions [26]. Previous studies also reported decreased thresholds for the other four substances at elevated ethanol concentrations relative to lower concentrations. This trend diverges from the increased isopentanol threshold observed here, a discrepancy potentially attributable to inter-panel differences in odor sensitivity or methodological variations among studies [44].

#### 3.4.2. The Effect of Ethanol Concentration on the OT of Binary Mixtures of Alcohol and Esters

**Figure 3:**
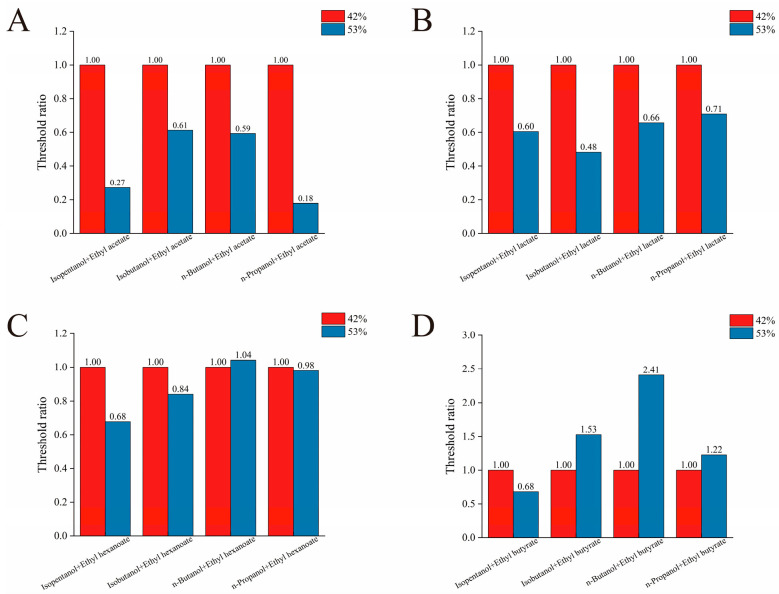
The effects of ethanol concentration on the OT of binary mixtures of four kinds of alcohol and ethyl acetate (**A**), ethyl lactate (**B**), ethyl hexanoate (**C**), and ethyl butyrate (**D**), respectively.

Then, the effect of ethanol concentration on the olfactory thresholds of alcohol–ester binary mixtures was investigated. Sixteen distinct binary alcohol–ester mixtures were prepared at precise concentrations. The thresholds obtained at 53% ethanol aqueous solution were then compared to those measured at 42% ethanol aqueous solution. As shown in Figure 3A, the ratio of the detection threshold in 53% ethanol aqueous solution to that in 42% ethanol aqueous solution for binary mixtures of isopentanol, isobutanol, n-butanol, and n-propanol with ethyl acetate is less than 1. Notably, this ratio falls below 0.5 for mixtures containing isobutanol or n-propanol with ethyl acetate. Figure 3B reveals a similar trend for binary mixtures of these four alcohols with ethyl lactate, where all ratios are less than 1. Notably, the threshold ratio for the isobutanol and ethyl lactate mixture exceeds this trend, falling below 0.5. Conversely, Figure 3C shows that the threshold ratio for the n-butanol and ethyl hexanoate binary mixture is greater than 1, indicating a marginally higher detection threshold at 53% ethanol aqueous solution compared to 42% ethanol aqueous solution. As shown in Figure 3D, the detection threshold ratio for the binary mixture of n-butanol and ethyl butyrate significantly exceeded the OT of 42% ethanol aqueous solution, reaching 2.41-fold that value. The threshold ratios for binary mixtures containing isobutanol and n-propanol with ethyl butyrate at the 53% ethanol aqueous solution were also higher than 42%, reaching 1.53 and 1.22 folds, respectively. All three of these binary mixtures exhibited threshold ratios greater than 1. Based on the above analysis, the four binary mixtures with threshold ratios exceeding 1 included n-butanol with ethyl hexanoate, isobutanol with n-butanol, and n-propanol with ethyl butyrate. Conversely, the ratio of three binary mixtures (isobutanol with ethyl acetate, n-propanol with ethyl acetate, and isobutanol with ethyl lactate) was below 0.5. Additionally, the threshold ratios of nine binary mixtures were between 0.5 and 1.

Currently, the literature exists on investigating the influence of ethanol concentration (specifically high vs. reduced alcohol content) on individual substance OT. Studies have determined the aroma thresholds of 87 aroma compounds in both 40% and 50% ethanol aqueous solutions using OTs [26]. Currently, no published studies specifically address how high and low ethanol concentrations influence the OT of binary mixtures. This study reveals a critical phenomenon that high alcohol content elevates the thresholds of most individual compounds yet reduces those of the binary mixtures, suggesting an odor enhancement mechanism. Consequently, alterations in mixture thresholds induced by alcohol are likely to significantly influence the varying sensory profiles observed at different alcohol concentrations by affecting perceptual sensitivity. This study provides a scientific basis for the flavor regulation of light-aroma Baijiu with different alcohol contents. Specifically, the consistency of product flavor can be improved by optimizing ethanol concentration or adjusting the ratio of key ester and alcohol compounds.

### 3.5. The Effect of Ethanol Concentration on the Interaction of Binary Mixtures of Alcohol and Esters

Given that ethanol concentration influences the OT of alcohol–ester binary mixtures, further research will investigate whether alcohol concentration modulates mixture interactions. Consequently, we will examine the perception of substance aromas across varying alcohol concentrations from an interaction perspective based on S-curve and OAV.

#### 3.5.1. Analysis of Interactions Between Binary Mixtures Based on S-Curve

**Figure 4:**
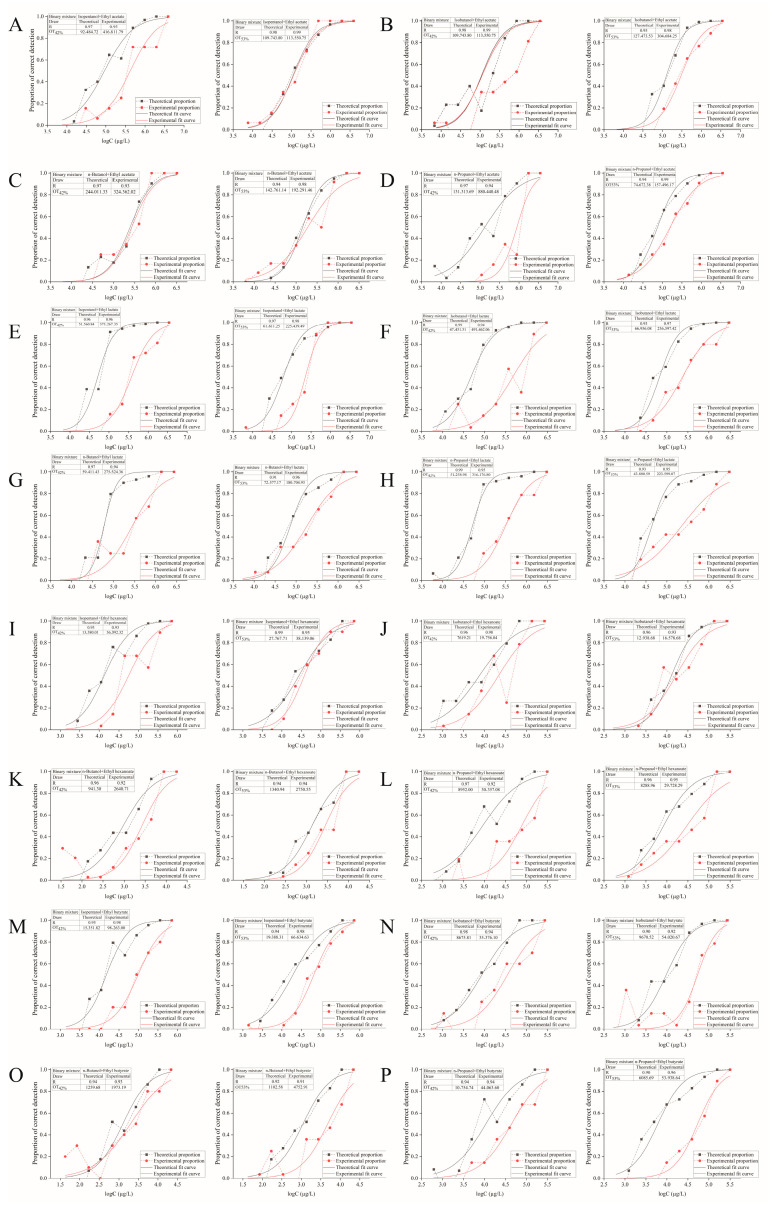
S-shaped curves for binary mixtures of ethanol aqueous solutions (42% and 53%) with various alcohols and esters. (**A**) Isopentanol + ethyl acetate; (**B**) Isobutanol + ethyl acetate; (**C**) n-Butanol + ethyl acetate; (**D**) n-Propanol + ethyl acetate; (**E**) Isopentanol + ethyl lactate; (**F**) Isobutanol + ethyl lactate; (**G**) n-Butanol + ethyl lactate; (**H**) n-Propanol + ethyl lactate; (**I**) Isopentanol + ethyl hexanoate; (**J**) Isobutanol + ethyl hexanoate; (**K**) n-Butanol + ethyl hexanoate; (**L**) n-Propanol + ethyl hexanoate; (**M**) Isopentanol + ethyl butyrate; (**N**) Isobutanol + ethyl butyrate; (**O**) n-Butanol + ethyl butyrate; (**P**) n-Propanol + ethyl butyrate.

As shown in Table 4 and Figure 4, D values for all 16 binary mixtures exceeded 1 at both ethanol concentrations tested. This systematically indicates a masking effect between alcohol and ester compounds. Studies on Oolong tea by Zhu et al. [45] suggest that compounds with distinct structures or aroma profiles often exhibit mutual masking effects. This also aligns with findings that the four binary mixtures formed by n-propanol paired with ethyl acetate, ethyl lactate, ethyl hexanoate, or ethyl butyrate demonstrate masking interactions [15]. Furthermore, the D values for most binary mixtures were higher in 42% ethanol aqueous solution than in 53% ethanol aqueous solution, indicating stronger masking effects between components at the lower ethanol concentration. This is consistent with previous studies where the threshold was higher than 53% in a 42% ethanol aqueous solution.

Among the 16 binary mixtures tested at two alcohol concentrations (42% and 53% ethanol aqueous solutions), all exhibited a masking effect, with the masking intensity generally weaker in the 53% ethanol aqueous solutions: 12 out of the 16 mixtures showed lower D-values (an indicator of masking effect strength) compared to the 42% ethanol aqueous solutions. These 12 mixtures included binary mixture of isopentanol, isobutanol, or n-propanol with ethyl acetate; isopentanol, isobutanol, n-propanol, or n-butanol with ethyl lactate; isopentanol, isobutanol, or n-butanol with ethyl hexanoate; and isopentanol or n-propanol with ethyl butyrate. Within the 53% ethanol aqueous solution, the isopentanol-ethyl acetate mixture exhibited the lowest D-value (1.03). To date, only a limited number of studies in the literature have investigated differences in the threshold values of individual substances under varying ethanol concentrations; few studies have examined the interactions of binary mixtures across different ethanol concentrations. Therefore, the results of this study indicate that ethanol concentration also influences the interactions between aroma-active compounds.

#### 3.5.2. Analysis of Interactions Between Binary Mixtures Based on OAV

The OAV method has been widely applied to binary, ternary, or more complex mixture systems [46,47,48]. Generally, a larger X-value is indicative of a higher degree of masking. As shown in Table 5, all binary mixtures exhibited a masking effect in two ethanol aqueous solutions with volume fractions of 42% and 53%. Notably, the masking intensity was generally lower in the 53% ethanol aqueous solutions—9 out of the 16 mixtures had lower X-values compared to those in the 42% ethanol aqueous solutions. These 9 mixtures specifically include: binary mixtures of ethyl acetate with isopentanol, isobutanol, n-butanol, or n-propanol; binary mixtures of ethyl lactate with isopentanol or isobutanol; the binary mixture of n-propanol and ethyl hexanoate; and binary mixtures of ethyl butyrate with isopentanol or isobutanol. Furthermore, the isopentanol-ethyl acetate mixture showed the lowest X-value (1.02) in the 53% ethanol aqueous solution, which is consistent with the variation trend of the D-values. This finding suggests that these compounds influence the perception of overall flavor through a masking mechanism. Niu et al. [49] reported similar findings in their study on ester-ester interactions in QXB. In contrast, compounds with distinct structural features exert a masking effect. Notably, the experimental results regarding the masking effect derived from the S-curve method are consistent with those from the OAV method, suggesting that the masking effect may arise from the structural differences between alcohol–ester pairs. Therefore, the modulation of flavor interactions by ethanol concentration and the masking effects among key aroma compounds in QXB offer actionable insights for industrial applications, including optimizing Baijiu production processes and guiding the development of targeted flavor adjustment strategies.

### 3.6. Verification Experiment: The Masking Effect of Alcohol–Ester Binary Mixtures Is Weaker in Higher Concentration Ethanol Aqueous Solutions

**Figure 5:**
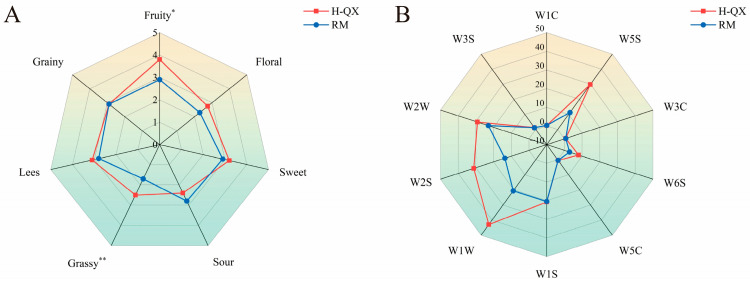
Comparison of aroma profiles between H-QX and RM based on radar chart of sensory evaluation (**A**) and E-nose (**B**). *, significant difference (*p* < 0.05); **, highly significant difference (*p* < 0.01).

To confirm that the interactions between alcohol–esters vary in different alcohol–water solutions, sensory evaluation experiments were carried out on Baijiu samples with the same main alcohol–ester concentration of two different ethanol aqueous solutions. As shown in Figure 5A, the H-QX sample exhibited higher aroma scores for floral, fruity, sweet, grainy, and grassy compared to the RM. Among these, significant differences were observed between the two samples in fruity and grassy aromas. This indicates that the masking effect of alcohols and esters is weaker in higher-alcohol samples than in lower-alcohol ones, suggesting that a higher alcohol content promotes the release of alcoholic and ester aroma compounds—a finding consistent with previous conclusions. As can be seen in Figure 5B, the electronic nose responses for sensors W1W, W2W, W2S, and W5S were higher in the H-QX sample than in the RM sample, indicating that changes in alcohol content have a notable influence on the response values of these electronic nose sensors.

## 4. Conclusions

This study systematically clarifies the regulatory role of ethanol concentration in the flavor expression of QXB through integrated physicochemical analysis, sensory evaluation, and modern analytical techniques. The results showed that the content of key flavor-related substances in H-QX was higher, the total acid content decreased, and the total ester content increased. H-QX exhibits more prominent fruity, grassy, and grainy aromas, whereas L-QX has stronger sour and lees notes. Ethanol concentration also modulates the olfactory thresholds of core alcohols and esters, as well as the interaction between alcohol–ester binary mixtures—all tested mixtures show masking effects, with more pronounced masking in reduced-alcohol systems, which is a key factor leading to weaker aroma expression in reduced-alcohol QXB. Increasing ethanol concentration can mitigate these masking effects, thereby enhancing the release of key aroma attributes.

This study has certain limitations: it focuses on only two ethanol concentrations and eight key alcohol–ester compounds, lacking exploration of other flavor-active substances (such as aldehydes and organic acids) and more ethanol gradients. Future research will expand the scope to include more ethanol concentrations and flavor compounds and combine molecular docking to explore the binding mechanism between key flavor compounds and olfactory receptors, providing a deeper molecular-level explanation for flavor differences. From a practical perspective, this study holds important guiding significance for the development of reduced-ethanol beverages (including reduced-alcohol QXB). It emphasizes that when developing such products, full consideration must be given to how interactions between flavor substances alter perceptual thresholds and thus affect final flavor. For reduced alcohol QXB or similar reduced-ethanol drinks, targeted strategies—such as adjusting ethanol concentration to an appropriate range to mitigate masking effects or supplementing key flavor compounds to compensate for aroma loss—can effectively solve the common “insufficient aroma” problem in reduced alcohol products. This not only provides a scientific basis for flavor regulation in the production of reduced-ethanol QXB but also offers a reference for the development of other high-quality reduced-ethanol beverages that balance consumer health needs and sensory experience, aligning with the industry trend toward low-to-medium alcohol content and high quality.

## Figures and Tables

**Table 1:** Definition of aroma properties and reference samples.

Aroma Attribute	Definition	Reference Sample	Score
Floral	Floral aroma similar to fresh flowers in baijiu	2.89 mg/L phenethyl alcohol in 50% ethanol-water solution	5
Fruity	Fruity aroma similar to ripe fruits in baijiu	8.15 mg/L ethyl butyrate in 50% ethanol-water solution	5
Sweet	Sweet aroma similar to honey and sweet fruits in baijiu	9.1 mg/L γ-Nonalactone in 50% ethanol-water solution	5
Grassy	The grassy aroma in Baijiu	2 mg/L hexanal in a 50% ethanol aqueous solution	5
Grainy	Aroma presented after fermentation and distillation of grain raw materials	20 g sorghum steamed in boiling water for 30 min	5
Lees	Aroma characteristic similar to fermented distillers’ grains in baijiu	Distillers’ grains	5
Sour	Sour aroma characteristic similar to rotten fruits and vinegar in baijiu	Aged vinegar	5

**Table 2:** Physicochemical analysis of QXB.

Physicochemical Indices	L-QX	H-QX
Alcohol content (%)	41.90 ± 0.08	52.90 ± 0.08
Total acids (g/L)	1.48 ± 0.01	1.20 ± 0.04
Total esters (g/L)	1.52 ± 0.02	2.05 ± 0.00

**Table 3:** Content of major volatile compounds in two QXB samples.

No.	Compound	CAS	Calibration Equations	L-QX (mg/L)	H-QX (mg/L)
	Esters				
A1	Ethyl formate	109-94-4	y = 0.4893x + 0.1611	ND	5.59 ± 0.13 b
A2	Ethyl acetate	141-78-6	y = 0.7176x − 2.4239	1043.94 ± 4.06 a	1580.04 ± 0.01 b
A3	Ethyl butyrate	105-54-4	y = 1.1186x + 0.2588	1.15 ± 0.05 a	5.69 ± 0.85 b
A4	Ethyl isovalerate	108-64-5	y = 2.0679x + 0.113	ND	0.3 ± 0.01 b
A5	Ethyl 2-methylbutanoate	7452-79-1	y = 1.0497x + 0.5285	ND	2.69 ± 0.24 b
A6	Isoamyl acetate	123-92-2	y = 1.1373x − 0.0158	ND	3.46 ± 0.01 b
A7	Ethyl valerate	539-82-2	y = 1.0635x + 0.2343	ND	6.2 ± 0.15 b
A8	Ethyl hexanoate	123-66-0	y = 1.2155x + 0.1776	2.03 ± 0.01 a	3.66 ± 0.16 b
A9	Ethyl lactate	97-64-3	y = 0.6042x + 5.3709	935.15 ± 3.83 a	1362.66 ± 6.50 b
A10	Ethyl octanoate	106-32-1	y = 1.2534x + 0.0205	ND	3.02 ± 0.07 b
A11	Ethyl decanoate	110-38-3	y = 0.2538x + 0.098	ND	6.36 ± 0.03 b
A12	Ethyl nonanoate	123-29-5	y = 1.3123x + 0.0169	ND	8.66 ± 0.47 b
A13	Ethyl benzoate	93-89-0	y = 1.3057x + 0.0561	5.56 ± 0.04 a	7.71 ± 0.06 b
A14	Ethyl leucinate	868-27-5	y = 1.4736x − 0.281	1.7 ± 0.13 a	2.01 ± 0.14 b
A15	Diethyl malonate	105-53-3	y = 1.2578x − 0.3274	0.94 ± 0.01 a	1 ± 0.01 b
A16	Diethyl succinate	123-25-1	y = 1.6106x − 0.2499	1.13 ± 0.18 a	1.41 ± 0.01 a
A17	Ethyl tridecanoate	28,267-29-0	y = 3.2391x − 0.3713	0.43 ± 0.01 a	0.5 ± 0.01 b
	Alcohols				
B1	Methanol	67-56-1	y = 0.5661x + 3.995	46.46 ± 1.41 a	37.58 ± 3.21 b
B2	n-Propanol	71-23-8	y = 1.0113x + 0.5785	103.9 ± 0.83 a	152.17 ± 1.59 b
B3	Isobutanol	78-83-1	y = 1.1662x + 0.4051	96.41 ± 0.48 a	130.48 ± 0.29 b
B4	n-Butanol	71-36-3	y = 0.2577x + 0.0066	3.55 ± 0.15 a	5.17 ± 0.11 b
B5	Active amyl alcohol	137-32-6	y = 0.2549x − 0.6288	42.76 ± 0.01 a	62.77 ± 0.29 b
B6	Isopentanol	123-51-3	y = 1.2562x + 0.4895	251.43 ± 1.50 a	352.93 ± 0.94 b
B7	n-Hexanol	111-27-3	y = 0.2637x − 0.0263	2.01 ± 0.17 a	2.73 ± 0.24 b
B8	2,3-Butanediol	513-85-9	y = 0.7996x − 0.7173	7.15 ± 0.23a	8.07 ± 0.20 b
B9	2-Phenylethanol	60-12-8	y = 1.4761x + 0.3748	3.3 ± 0.23 a	4.07 ± 0.05 b
	Acids				
C1	Acetic acid	64-19-7	y = 0.3938x − 9.5445	497.58 ± 6.71 a	630.81 ± 0.18 b
C2	Propionic acid	79-09-4	y = 0.5853x − 0.7433	ND	13.68 ± 1.38 b
C3	Isobutyric acid	79-31-2	y = 0.8139x − 0.0934	7.34 ± 0.63 a	8.84 ± 0.37 b
C4	Isovaleric acid	503-74-2	y = 0.9433x − 0.0793	ND	5.27 ± 0.50 b
C5	Lactic acid	50-21-5	y = 0.6465x − 1.4017	660.42 ± 0.16 a	620.22 ± 0.09 b
	Ketones				
D1	Acetoin	513-86-0	y = 0.8023x + 0.1368	5.5 ± 0.03 a	4.86 ± 0.21 b
	Aldehydes				
E1	Acetaldehyde	75-07-0	y = 0.4234x + 2.0894	123.92 ± 0.96 a	138.87 ± 1.46 b
E2	Acetal	105-57-7	y = 0.2216x − 0.7878	91.35 ± 0.53 a	102.38 ± 2.22 b
E3	Isobutyraldehyde	78-84-2	y = 0.0426x − 0.0449	9.07 ± 0.28 a	20 ± 0.64 b
E4	Furfural	98-01-8	y = 0.8353x + 3.0736	20.06 ± 0.95 a	19.37 ± 0.85 a

Note: Values in the same row with different letters (a, b) indicate that they are significantly different at *p* < 0.05; ND means not detected.

**Table 4:** D-values of alcohol–ester binary mixtures at different alcohol concentrations.

No.	Binary Mixture	42%	Interaction Relationship	53%	Interaction Relationship
		D		D	
1	Isopentanol + Ethyl acetate	4.51	Masking	1.03	Masking
2	Isobutanol + Ethyl acetate	3.48	Masking	2.39	Masking
3	n-Butanol + Ethyl acetate	1.33	Masking	1.35	Masking
4	n-Propanol + Ethyl acetate	6.70	Masking	2.11	Masking
5	Isopentanol + Ethyl lactate	7.24	Masking	3.66	Masking
6	Isobutanol + Ethyl lactate	10.36	Masking	3.53	Masking
7	n-Butanol + Ethyl lactate	4.64	Masking	2.49	Masking
8	n-Propanol + Ethyl lactate	6.17	Masking	5.10	Masking
9	Isopentanol + Ethyl hexanoate	4.15	Masking	1.37	Masking
10	Isobutanol + Ethyl hexanoate	2.59	Masking	1.28	Masking
11	n-Butanol + Ethyl hexanoate	2.81	Masking	2.05	Masking
12	n-Propanol + Ethyl hexanoate	3.39	Masking	3.59	Masking
13	Isopentanol + Ethyl butyrate	6.40	Masking	3.44	Masking
14	Isobutanol + Ethyl butyrate	4.08	Masking	5.59	Masking
15	n-Butanol + Ethyl butyrate	1.57	Masking	4.31	Masking
16	n-Propanol + Ethyl butyrate	4.10	Masking	8.86	Masking

**Table 5:** X-values of alcohol–ester binary mixtures at different alcohol concentrations.

No.	Binary Mixture	42%	Interaction Relationship	53%	Interaction Relationship
		X		X	
1	Isopentanol + Ethyl acetate	2.54	Masking	1.02	Masking
2	Isobutanol + Ethyl acetate	5.69	Masking	2.80	Masking
3	n-Butanol + Ethyl acetate	2.21	Masking	2.02	Masking
4	n-Propanol + Ethyl acetate	2.97	Masking	1.52	Masking
5	Isopentanol + Ethyl lactate	1.25	Masking	1.07	Masking
6	Isobutanol + Ethyl lactate	3.33	Masking	1.40	Masking
7	n-Butanol + Ethyl lactate	1.05	Masking	1.21	Masking
8	n-Propanol + Ethyl lactate	1.14	Masking	1.24	Masking
9	Isopentanol + Ethyl hexanoate	1.34	Masking	1.49	Masking
10	Isobutanol + Ethyl hexanoate	1.04	Masking	1.04	Masking
11	n-Butanol + Ethyl hexanoate	1.08	Masking	1.17	Masking
12	n-Propanol + Ethyl hexanoate	1.31	Masking	1.17	Masking
13	Isopentanol + Ethyl butyrate	3.44	Masking	3.29	Masking
14	Isobutanol + Ethyl butyrate	4.37	Masking	5.03	Masking
15	n-Butanol + Ethyl butyrate	4.20	Masking	5.12	Masking
16	n-Propanol + Ethyl butyrate	3.51	Masking	2.48	Masking

## Data Availability

The original contributions presented in this study are included in the article/Supplementary Materials. Further inquiries can be directed to the corresponding authors.

## Supplementary Materials

The following supporting information can be downloaded at: https://www.mdpi.com/article/10.3390/foods14244290/s1, The proof of ethical approval was shown in Table S1.

## Abbreviations

The following abbreviations are used in this manuscript:

OT	Olfactory threshold
OAV	Olfactory threshold
QXB	Qingxiangxing Baijiu
NXB	Nongxiangxing Baijiu
JXB	Jiangxiangxing Baijiu
GC-FID	Gas chromatography flame ionization detection
HPLC	High-performance liquid chromatography
3-AFC	Three-alternative forced-choice
L-QX	Baijiu with alcohol contents of 42% (Reduced alcohol)
H-QX	Baijiu with alcohol contents of 53% (Hight alcohol)
QDA	Quantitative descriptive analysis
E-nose	Electronic nose
RM	Recomposed model
