# Supplementary material for: Impact of Alcohol Content on Alcohol–Ester Interactions in Qingxiangxing Baijiu Through Threshold Analysis
Source: Foods. 2025 Dec 12;14(24):4290. doi: 10.3390/foods14244290 (PMC12732988; doi:10.3390/foods14244290)
Supplement: Supplementary file 1 [file foods-14-04290-s001.zip › Table S1.pdf]

Ethics Review Form of the Scientific Research Ethics Committee of Tianjin University of Science and Technology

Application date：

<table>
<tr><td colspan="3">Project name：Impact of Alcohol Content on Alcohol-Ester Interactions in Qingxiangxing Baijiu through Threshold Analysis</td></tr>
<tr><td rowspan="2">Project leader：Liping Du</td><td>Title：Professor</td><td>Institution：Tianjin University of Science and Technology</td></tr>
<tr><td>Tel：+86 022-60602723</td><td>E-mail：dlp123@tust.edu.cn</td></tr>
<tr><td colspan="3">Review type：☑ Application project ☐Post-approval project ☐Continuation project ☐ Entrusted project</td></tr>
<tr><td colspan="3">Project source：Tianjin Postgraduate Scientific Research Innovation Project.</td></tr>
<tr><td colspan="3">Review data

☑Experimental scheme ☑Informed consent ☐Other information
Including: safety data of test supplies, qualification certificate of production enterprise, qualification certificate of test supplies provider.</td></tr>
<tr><td colspan="3">Summary of research content and research program involving ethics
Ten panelists (2 males and 8 females, ranging in age from 22 to 36, an average of 25) with experience in olfactory experiments and quantitative descriptive analysis were recruited from our laboratory. Before participating in the experiment, they were told about the experimental scheme and were asked about basic information, willingness and interest to join the group and their health status. All participants received written information about the study, and they signed informed consent to participate. Each panelist has received no less than 90 h of good training over three months, which lasts for one semester. The specific evaluation process is as follows: baijiu samples (15 mL each) were poured into a 50 mL national standard wine tasting glass, and olfactory score was determined. Sensory evaluation of baijiu was classified into 7 attributes including floral, fruity, sweet, grassy, grainy, lees, and sour. The panelist smelt the samples and gave aroma intensity of the samples. The intensities of the odor descriptors were rated using a</td></tr>
</table>

scale from 0 to 5, where 0 meant none or imperceptible intensity, 5 meant high intensity. Each sample was evaluated three times by each panelist.

The olfactory detection thresholds of the key aroma compounds (when present individually) and the measured olfactory detection thresholds of the pairwise binary mixtures were determined using the 3-AFC method, which was performed in accordance with the ISO 13301:2018 standard. The sensory panel consists of 15 judges (9 females and 6 males, aged 22-25 years). They were told about the experimental scheme and were asked about basic information, willingness and interest to join the group and their health status. Prior to the formal experiment, they received 2 h professional training five times a week for three months. Each sample was evaluated three times by each panelist.

Ethics Committee reviews comments

Upon review, the research content and plan of the project fully take into account the principles of safety and fairness, the three principles of substitution, reduction and optimization during the test. This study obtained the consent of the subjects, protect the rights and interests of animals or people and minimize the pain, hurt and tension of animals. and fully protect the emotional consent of test subjects. There is no conflict of interest between research content and research results.

Ethics Committee of Tianjin University of Science and Technology

Date:
